# A Multimodal Deep Learning Approach for Legal English Learning in Intelligent Educational Systems

**DOI:** 10.3390/s25113397

**Published:** 2025-05-28

**Authors:** Yanlin Chen, Chenjia Huang, Shumiao Gao, Yifan Lyu, Xinyuan Chen, Shen Liu, Dat Bao, Chunli Lv

**Affiliations:** 1 China Agricultural University, Beijing 100083, China; 2 Faculty of Education, Monash University, Melbourne, VIC 3800, Australia; 3 Faculty of Humanities, China University of Political Science and Law, Beijing 102249, China; 4 School of Informational Technology and Management, University of International Business and Economics, Beijing 100029, China; 5 Law School, University of International Relations, Beijing 100091, China; 6 National School of Development, Peking University, Beijing 100871, China

**Keywords:** visual and acoustic sensor integration, multimodal semantic fusion, human-centered intelligent education, vision–language–speech unified encoding

## Abstract

With the development of artificial intelligence and intelligent sensor technologies, traditional legal English teaching approaches have faced numerous challenges in handling multimodal inputs and complex reasoning tasks. In response to these challenges, a cross-modal legal English question-answering system based on visual and acoustic sensor inputs was proposed, integrating image, text, and speech information and adopting a unified vision–language–speech encoding mechanism coupled with dynamic attention modeling to effectively enhance learners’ understanding and expressive abilities in legal contexts. The system exhibited superior performance across multiple experimental evaluations. In the assessment of question-answering accuracy, the proposed method achieved the best results across BLEU, ROUGE, Precision, Recall, and Accuracy, with an Accuracy of 0.87, Precision of 0.88, and Recall of 0.85, clearly outperforming the traditional ASR+SVM classifier, image-retrieval-based QA model, and unimodal BERT QA system. In the analysis of multimodal matching performance, the proposed method achieved optimal results in Matching Accuracy, Recall@1, Recall@5, and MRR, with a Matching Accuracy of 0.85, surpassing mainstream cross-modal models such as VisualBERT, LXMERT, and CLIP. The user study further verified the system’s practical effectiveness in real teaching environments, with learners’ understanding improvement reaching 0.78, expression improvement reaching 0.75, and satisfaction score reaching 0.88, significantly outperforming traditional teaching methods and unimodal systems. The experimental results fully demonstrate that the proposed cross-modal legal English question-answering system not only exhibits significant advantages in multimodal feature alignment and deep reasoning modeling but also shows substantial potential in enhancing learners’ comprehensive capabilities and learning experiences.

## 1. Introduction

With the rapid development of artificial intelligence and intelligent sensor technologies, language learning is gradually shifting towards a multimodal, interactive approach [1]. This shift is particularly important in legal English education. Legal English, being a highly specialized field, requires learners to master complex legal terminology, rigorous logical structures, and extensive case analysis skills [2]. Moreover, legal English learning not only involves language proficiency but also requires learners to understand and express various forms of evidence and contexts [3]. In real-world legal work, case analysis often relies not only on textual materials but also on a vast array of evidence such as images, audio recordings, and other multimodal information [4]. This makes legal English learning content diverse and complex [5]. Traditional systems predominantly rely on text input and static exercises and lack systematic training in the semantic correlations between language and visual evidence, which makes it difficult for students to effectively apply legal language in real-life scenarios [6]. The traditional legal English teaching methods mainly rely on text-based learning and case analysis [7]. Through textbooks, legal texts, and case studies, students are able to acquire some basic legal knowledge and expressive skills [8]. However, this single-modality text-based approach has significant limitations. First, most of the learning content in traditional methods is based on written materials, with insufficient use of evidence types such as case images, videos, or audio [9]. In real legal practice, case judgments often depend on multiple sources of evidence, not just written text. Visual materials (e.g., photos of crime scenes, evidence images) and audio information (e.g., courtroom debates, witness testimonies) are essential components of legal analysis [10]. Traditional systems cannot effectively process these diverse input types, leaving learners unable to integrate and analyze these multimodal inputs when faced with complex cases [11]. Furthermore, traditional legal English education often remains static, with students only improving their language skills through reading and writing exercises, lacking opportunities for interaction in real-world contexts [12]. In actual legal work, learners need to understand complex legal texts and quickly respond in dynamic legal environments [13]. Lastly, traditional teaching methods offer limited interaction between students and the system, preventing learners from receiving timely feedback on their responses and lacking comprehensive evaluation of their responses. This results in difficulties in improving legal reasoning and language expression in practical applications [14].

The study of legal English represents a highly specialized task, requiring learners to simultaneously master semantic comprehension, evidentiary interpretation, and precise legal expression. These demands inherently involve multimodal information, including text, images, and speech, which poses significant challenges to traditional text-centered pedagogical methods due to their limited capacity for processing heterogeneous legal inputs such as courtroom imagery or spoken testimony. Although deep learning has opened new avenues for this domain [15], and recent advances in natural language processing (NLP), computer vision (CV), and speech recognition have enabled the integration of multimodal data into legal education [16], several limitations persist. Models such as BERT, CLIP, and wav2vec have demonstrated effectiveness in processing legal texts, aligning images with text, and transcribing speech into text, respectively [17,18]. However, their application in legal English instruction continues to encounter unresolved issues, including limited precision in cross-modal alignment, difficulty in conveying fine-grained semantic nuances across modalities, and the absence of real-time interactive feedback mechanisms. Prior research has highlighted the potential of cross-modal approaches, such as audio–text fusion for event detection [19], multimodal knowledge graph construction [20], and joint modeling of speech and text for emotion recognition [21,22], yet few studies have adequately addressed the distinctive challenges presented by legal discourse.

**Figure 1:**
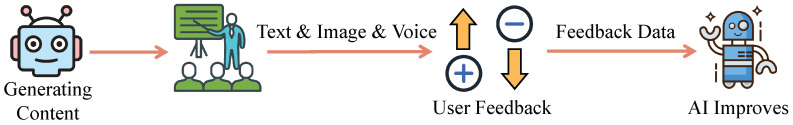
Illustration of the structure of multimodal feedback data composed of text, image, and voice signals that are collected and integrated to form the basis of sensor-enhanced intelligent education systems. The “+” symbol denotes the semantic integration of heterogeneous modalities, aiming to enrich learner understanding through complementary cues; the “−” symbol represents modal inconsistencies or interference, which the system resolves via alignment or correction strategies during multimodal fusion.

To address these challenges, a cross-modal question answering system for legal English, integrating image and audio sensor data, is proposed. This system leverages deep learning techniques across visual, textual, and speech modalities to construct an intelligent interactive platform applicable to diverse legal English education scenarios. It aims to facilitate the joint training of case comprehension and legal language expression in realistic legal contexts, as illustrated in Figure 1. The system acquires visual evidence and speech input through cameras and microphones, which are then processed via a structured three-stage pipeline. First, case images and legal questions are jointly encoded by a visual-language module. A CNN-Transformer hybrid network is employed to extract fine-grained visual features, which are fused with the Sentence-BERT-encoded question vectors through a cross-modal attention mechanism, yielding a unified semantic representation. Simultaneously, learners’ spoken responses are processed by a speech recognition and semantic parsing module. This module utilizes a Transformer-based ASR model to transcribe the audio into text, which is subsequently interpreted by a diffusion Transformer into semantically enriched legal representations, ensuring coherence across temporal and modal contexts. Finally, the question–answer matching and feedback module integrates these multimodal features and employs a large-scale vision–language–speech fusion model to assess the quality of responses. A semantic alignment score is computed between the learner’s input and the reference answer, and adaptive feedback is generated based on attention activations and similarity thresholds. Through this pipeline, raw sensor data are transformed into structured and interpretable legal language representations, enabling an interactive, context-aware legal English learning experience grounded in multimodal comprehension. The specific innovations introduced include:

1. Deep fusion of multimodal data: This system is the first to integrate data from image, speech, and text modalities for legal English question answering, extracting and understanding legal information from images and speech through deep learning models, breaking the limitations of traditional text-only input systems, and providing richer and more comprehensive learning materials.
2. Cross-modal question-answering system: The system not only understands the semantic relationship between images and text but also allows legal question answering through speech input, incorporating image evidence for comprehensive analysis. Users interact with the system through speech or image inputs, and the system provides real-time feedback and scoring based on the learner’s responses, helping them better master the use of legal English.
3. Multimodal semantic fusion model: The system integrates CLIP for image–text semantic alignment, a Transformer-based ASR model for speech transcription and parsing, and Sentence-BERT for deep legal question encoding. On this basis, we propose a unified vision–language–speech embedding framework that supports fine-grained semantic integration across modalities. Furthermore, an affordance-driven masking mechanism and multi-level alignment strategy are introduced to enhance the model’s focus on legally relevant information, enabling more accurate reasoning and feedback in complex legal scenarios.
4. Real-world scenario simulation and teaching feedback: By integrating image and speech sensors, the system simulates real courtroom scenarios or case analysis tasks, providing a more practical interactive learning experience. In interacting with the system, learners not only improve their legal English expression abilities but also enhance their legal thinking in real-world legal contexts.

## 2. Related Work

### 2.1. Multimodal Semantic Understanding Technologies

Compared with traditional text-only understanding approaches, multimodal semantic understanding integrates image, text, and audio modalities, demonstrating significant advantages in information completeness, adaptability to heterogeneous inputs, and the realization of contextualized and interactive learning in educational scenarios. These advantages provide a more effective technical pathway for enhancing language transferability and semantic depth [23]. Recent advances in image–text joint learning (e.g., VisualBERT [24]) and speech–text alignment (e.g., wav2vec [25]) have substantially extended the applicability of natural language processing (NLP) in multimodal tasks [26]. These technologies enable systems to extract and align multimodal information, thereby improving the accuracy of semantic understanding [27]. For instance, VisualBERT extracts features and positional encodings from images and textual inputs using a pretrained CNN and a BERT encoder, respectively. The two modalities are then projected into a shared semantic space [28,29] and subsequently aligned via attention mechanisms, as formulated below:

(1) $$\begin{array}{cc} v & =f_{\mathrm{CNN}}\left(\mathrm{Resize}\left(I\right)\right),\;v\in R^{d_{v}}, \end{array}$$

(2) $$\begin{array}{cc} t & =f_{\mathrm{BERT}}\left(\mathrm{Tokenizer}\left(L\right)\right),\;t\in R^{d_{t}}, \end{array}$$

(3) $$\begin{array}{cc} v^{\prime } & =W_{v}v+b_{v},\;t^{\prime }=W_{t}t+b_{t}, \end{array}$$

(4) $$\begin{array}{cc} z & =\mathrm{Attention}\left(v^{\prime },t^{\prime }\right),\;z\in R^{d_{z}}, \end{array}$$

where I and L denote the input image and text, respectively; $f_{\mathrm{CNN}}$ and $f_{\mathrm{BERT}}$ represent the image and text encoders; $W_{v}$ and $W_{t}$ are linear projection matrices; Attention(·) denotes the cross-modal attention alignment operation; and z is the final joint semantic representation. However, the static nature of features in VisualBERT limits its ability to model dynamic relations between visual details and textual semantics, rendering it less effective in handling image quality variability and semantic heterogeneity in real educational environments. CLIP further extends this framework by employing contrastive learning to optimize image–text matching within a shared feature space [30,31]. The contrastive loss is defined as:

(5) $$L=-\sum_{i}\log\frac{\exp(v_{i}\cdot t_{i}/\tau )}{\sum_{j}\exp\left(v_{i}\cdot t_{j}/\tau \right)},$$

where τ is the temperature parameter and $v_{i}$ and $t_{j}$ represent the image and text feature vectors, respectively. This method enhances semantic consistency across modalities, thereby improving the accuracy of image–text retrieval tasks [32]. Despite its generalizability owing to large-scale training, CLIP lacks task-specific fine-tuning and thus exhibits limitations in recognizing domain-specific semantics such as legal terminology and evidentiary content. In the speech–text domain, wav2vec learns robust speech representations from unlabeled audio data and maps raw waveforms into a feature space suitable for downstream transcription and semantic tasks [33,34], formalized as:

(6) S=wav2vec(X),

where X denotes the input speech signal. For legal English learning scenarios, wav2vec enables accurate speech-to-text conversion and serves as a frontend for downstream semantic parsing pipelines [35]. However, it remains sensitive to speech variability such as accent and rate and provides only low-level acoustic features, which are insufficient for supporting high-level semantic reasoning and task-specific interpretation. Extended models such as SpeechBERT incorporate BERT’s textual reasoning capabilities, with speech inputs to improve semantic alignment and parsing accuracy [36,37]. Nevertheless, these models are characterized by architectural complexity, high computational cost, and strong dependency on domain-specific corpora, which limits their scalability and responsiveness when deployed in practical educational systems.

In high-stakes educational domains such as legal English, models must not only achieve accurate semantic alignment but also ensure the traceability of reasoning paths and decision rationales. Current mainstream methods adopt end-to-end architectures with deeply nested neural layers, which, while powerful in representation, often exhibit “black-box” characteristics that obscure the influence of specific features on final outputs. Although some studies have introduced attention visualization and embedding space analysis to enhance transparency, these mechanisms remain insufficient to meet the demand for explicit explainability from instructors or learners. Therefore, the construction of multimodal models with structured interpretability and semantically layered representations is essential for ensuring the reliability and pedagogical applicability of such systems. This design motivation underpins the introduction of structural alignment mechanisms and semantic feedback modules in the proposed framework. Furthermore, in the “Results and Discussion” section, the proposed method is quantitatively compared with mainstream baselines across multiple metrics and error margins and statistical fluctuations are analyzed to validate its superiority in both accuracy and robustness.

### 2.2. Sensor-Supported Language Learning Systems

In the field of language learning, the integration of image and speech sensors has become a crucial tool for enhancing learning outcomes [38]. Speech scoring systems are among the most common applications, primarily using speech recognition technology to evaluate learners’ pronunciation and provide feedback [39]. These systems typically assess pronunciation accuracy by comparing the standard pronunciation with the learner’s pronunciation. The basic evaluation formula can be expressed as:

(7) $$\mathrm{Score}=\frac{1}{N}\sum_{i=1}^{N}D\left(y_{i},{\hat{y}}_{i}\right),$$

where $y_{i}$ represents the standard pronunciation, ${\hat{y}}_{i}$ represents the learner’s pronunciation, *N* is the number of evaluated speech units, and $D(y_{i},{\hat{y}}_{i})$ represents the distance metric between the standard and learner’s pronunciation. Common distance metrics include dynamic time warping (DTW) and Euclidean distance. This method allows the system to provide a score and specific improvement suggestions based on the differences between the learner’s and the standard pronunciation [40]. Image reading practice platforms typically use image recognition technology to assist learners in recognizing and understanding the information in images [41]. For legal English learning, such platforms can be used to recognize images of crime scenes, evidence materials, etc., and provide learners with related legal questions based on the image content [42]. This method helps learners better understand legal contexts and enhance their ability to perceive and judge case details. For example, the system automatically identifies and annotates key elements in the image, assisting learners in understanding the case background and evidence, leading to more accurate legal expression in subsequent question-answering tasks [43]. Although these sensor-supported systems have achieved good results in certain domains, they still have limitations in terms of task complexity and feedback granularity. For instance, in speech scoring systems, the evaluation mainly relies on pronunciation accuracy and fails to consider other language features such as grammar and semantics [44]. In image reading practice, although basic elements in images can be identified, the understanding of the underlying legal information in the image remains limited. Enhancing the adaptability of these systems in complex tasks, especially in the high-demand domain of legal English learning, remains a key challenge for future development [5].

### 2.3. Legal Language Education and Interactive Question-Answering Technologies

Legal language education, as a specialized form of language learning, requires learners to not only master basic legal terminology and grammar structures but also to possess strong legal reasoning and thinking skills [45]. Traditional legal English education methods typically focus on text reading and case analysis, but this approach often lacks a contextual learning experience, making it difficult to cultivate students’ ability to handle cases in real legal scenarios [46]. As a result, interactive question-answering systems have been widely used in legal language education in recent years, especially in mock courtrooms and case analysis training, as these systems provide instant feedback to learners, helping them improve their legal understanding and expression abilities [47]. The core principle of interactive question-answering systems is based on NLP technology, which automatically understands learners’ questions and generates appropriate answers [48]. Through multiple rounds of questioning, the system helps learners analyze cases, reason legal issues, and provide suitable legal advice. These systems typically establish a legal knowledge base to categorize and parse common legal issues [49]. With the development of deep learning technologies, many systems are now able to automatically understand complex legal problems and offer relevant solutions. For legal English learning, interactive question-answering systems help learners practice language skills in real legal contexts and improve their ability to handle complex legal issues [50]. However, existing interactive question-answering systems still face some challenges in handling complex legal issues. In particular, during legal English learning, systems need to understand not only legal terminology and case background but also multimodal information such as evidence materials [51]. Traditional question-answering systems often focus on a single modality (e.g., text) and lack effective handling of other sensory inputs such as images and speech [2]. Therefore, introducing multimodal information into legal question-answering systems and achieving effective alignment between modalities is a critical direction for improving system performance. The multimodal legal English question-answering system proposed in this paper is designed to address these issues by integrating image, speech, and text information, aiming to provide more comprehensive and accurate support for legal English learning. By incorporating deep learning models, the system can process the semantic relationships between different modality data, offering learners a richer learning experience.

## 3. Materials and Methods

### 3.1. Data Collection

The construction and use of datasets in AI-assisted legal applications—particularly those involving judicial or institutional scenarios—must adhere to emerging legal and regulatory frameworks that govern the access, provision, and processing of source data. The dataset presented in this work was developed in accordance with responsible data handling principles, including transparency, authorized public access, and the exclusion of sensitive or private content. These considerations align with ongoing discussions in regulatory frameworks such as the U.S. Federal Rules of Evidence and the EU’s General Data Protection Regulation (GDPR), which emphasize lawful sourcing, traceability, and privacy protection in AI-based legal systems.

A dataset targeting the multimodal legal English question-answering task was constructed, encompassing data from three modalities: images, text, and audio. The image data were primarily collected from publicly available online platforms, including the United States court record website (CourtListener), the well-known legal resource platform FindLaw, and Wikimedia Commons. Specifically, the collected image types include case scene photographs, evidence item images, and scanned copies of documents and contracts, with each category containing between 1000 and 2000 samples, as shown in Table 1. To ensure the quality and semantic relevance of the images, a uniform resolution not lower than 1024 × 768 pixels was adopted during the collection process, prioritizing images with clear annotations, complete scenes, and significant legal evidentiary value. All images were manually filtered and preliminarily annotated after collection, with annotations covering case types (such as criminal, civil, and intellectual property), image themes (such as evidence items and accident scenes), and key information regions, thereby ensuring high usability and contextual relevance within legal scenarios. In terms of data processing, all samples underwent a standardized preprocessing pipeline involving resolution normalization, noise filtering, and semantic-preserving augmentation strategies tailored to legal visual content. The quality of the samples was evaluated through both technical and semantic criteria, including clarity, annotation completeness, and relevance to legal contexts. To address the limitation in data volume and ensure robustness in diverse learning scenarios, an open-source dataset, MultimodalQA, was further incorporated as an auxiliary resource for experimentation. This dataset complements the in-house collection by providing a broader distribution of multimodal question–answer pairs, thereby enabling more comprehensive training and generalization analysis. Regarding textual data, the questions and standard answers were compiled from public case commentaries and question–answer style analyses published by the United States Supreme Court, FindLaw case database, and Harvard Law Review. Approximately 1500 pairs of questions and answers were collected, covering major fields such as criminal law, contract law, tort law, and intellectual property law. It should be noted that these textual question–answer pairs were constructed independently of visual or auditory modalities and thus do not include any associated images or video materials. During text processing, the characteristics of the legal question–answer genre were strictly followed, ensuring that the questions exhibited appropriate complexity (e.g., involving multi-step reasoning, fact summarization, and legal application), while the answers remained organized, logically coherent, and reflective of standard legal expression norms. Additionally, to enhance the educational applicability of the corpus, certain texts were moderately simplified, maintaining professionalism while lowering language complexity to facilitate learner comprehension and response. Audio data were collected through simulated learning scenarios, mainly using Blue Yeti noise-reduction microphones and associated acoustic sensor devices. The recording subjects included both native English speakers and advanced English learners, with a balanced gender distribution and an age range spanning from 20 to 55 years. To ensure diversity, participants exhibited varied accents, including American, British, and Southeast Asian English. Each audio sample corresponded to one question, with a duration controlled between 10 and 90 s. Recordings were conducted in a quiet, echo-free environment, and a consistent sampling rate of 16 kHz at 16-bit resolution was applied. To further enhance the dataset’s naturalness and robustness, the speech samples encompassed variations in speaking speed (ranging from slow to fast-paced delivery), intonation patterns (from flat to expressive), and degrees of formality in legal expression. Additionally, mild background noise simulations (e.g., soft environmental sounds) were introduced to simulate realistic acoustic conditions. In total, approximately 1200 high-quality audio recordings were compiled, providing a solid foundation for training and evaluating multimodal models under diverse and practical speech scenarios.

The current dataset is closely aligned with the terminological conventions, argumentative structures, and case organization typical of the Anglo-American common law system, which facilitates effective modeling within this legal context. However, such alignment may limit the system’s applicability to other legal traditions, such as civil law systems, where legal reasoning and textual formats differ significantly. Moreover, since the speech data were primarily collected from native English speakers, the ASR component may exhibit elevated error rates when processing inputs from non-native learners, thereby reducing its robustness and inclusiveness across diverse learner populations.

### 3.2. Data Preprocessing and Augmentation

In multimodal learning systems, data preprocessing and augmentation are crucial steps for improving model performance and robustness, particularly in image and audio data processing. Appropriate preprocessing and augmentation techniques can effectively enhance the model’s adaptability to input data. For image and audio data, preprocessing primarily aims to clean and normalize raw data, while data augmentation generates variations of the data to improve the model’s ability to generalize to different input conditions. In this study, all preprocessing procedures followed widely adopted standards in the computer vision and speech processing communities. Image data were handled using OpenCV-based pipelines, incorporating resolution normalization (≥1024 × 768), denoising via median filtering, and geometric transformations constrained by semantic region masks. Audio preprocessing adhered to standard speech recognition settings, including a 16 kHz sampling rate, 16-bit depth, and silence removal using amplitude-based thresholding. Feature extraction and augmentation were implemented using industry-standard libraries such as librosa and PyDub. These choices ensure that the data fed into the model meet both technical consistency and reproducibility requirements.

#### 3.2.1. Image Data Preprocessing and Augmentation

Image data preprocessing and augmentation are essential in CV tasks because the quality and content of images directly affect the model’s learning performance. Common image preprocessing operations include edge detection, resolution standardization, and noise filtering. These steps help the model better extract meaningful information from the image while reducing the interference from irrelevant factors. Edge detection is a commonly used technique in image preprocessing aimed at highlighting important features in an image, such as object contours or edges. Common edge detection algorithms include Sobel operator and Canny edge detection. The Sobel operator is a gradient-based method used to detect edges in images. The computation process can be expressed by the following formulas:

(8) $$G_{x}=\left[\begin{array}{ccc} -1 & 0 & 1\\ -2 & 0 & 2\\ -1 & 0 & 1 \end{array}\right]⊗I,$$

(9) $$G_{y}=\left[\begin{array}{ccc} -1 & -2 & -1\\ 0 & 0 & 0\\ 1 & 2 & 1 \end{array}\right]⊗I,$$

where $G_{x}$ and $G_{y}$ represent the horizontal and vertical gradients of the image, ⊗ denotes the convolution operation, *I* represents the input image, and the kernels are manually defined based on the classical Sobel operator, as this step is used for fixed edge enhancement during preprocessing rather than trainable feature extraction. By applying Sobel convolution to the image, edge information is obtained, enhancing the prominence of important structures in the image. Resolution standardization is another common preprocessing step. In multimodal learning systems, image inputs may come from different sources, leading to variations in resolution and size. To ensure consistent processing of image data, it is necessary to resize all images to a standard resolution. For example, for an input image I, it can be resized to a fixed size $I^{\prime }$ using interpolation methods:

(10) $$I^{\prime }=\mathrm{Resize}\left(I,\left(w,h\right)\right),$$

where Resize(I,(w,h)) refers to resizing the input image I to dimensions (w,h). Common interpolation methods include nearest-neighbor interpolation and bilinear interpolation. Noise filtering refers to removing irrelevant noise from images, typically accomplished using median filtering or mean filtering. Noise can affect the extraction of image features, leading to decreased model performance. Median filtering is a commonly used denoising method that replaces the target pixel with the median of its neighboring pixels to reduce the impact of noise. Its computation can be expressed as:

(11) $$I_{\mathrm{new}}\left(x,y\right)=\mathrm{median}\left({\left\{I\left(x+i,y+j\right)\right\}}_{i=-k,j=-k}^{k}\right),$$

where $I_{\mathrm{new}}\left(x,y\right)$ represents the denoised pixel value, {I(x+i,y+j)} denotes the neighboring pixels around the target pixel, and *k* is the size of the filter. Through this method, noise in the image can be effectively suppressed. In addition to these basic image preprocessing methods, data augmentation techniques such as rotation, cropping, flipping, and scaling are employed to generate diverse image variants, enhancing the model’s adaptability to different scenarios. To ensure semantic integrity during augmentation, a region protection mechanism is applied based on annotated evidential areas, where transformations are restricted to non-critical regions to avoid occluding or distorting legally relevant content. Furthermore, cropping operations are guided by a saliency-aware and boundary-constrained strategy, which preserves the interpretability of key visual elements. A consistency validation pipeline is also integrated before and after augmentation to assess both image quality and content fidelity, ensuring that the generated data remain legally interpretable and applicable for downstream model training. For example, image rotation helps the model better learn rotation-invariant features. For an image I, rotation can be represented as:

(12) $$I^{\prime }=\mathrm{Rotate}\left(I,\theta \right),$$

where θ is the rotation angle and Rotate(I,θ) denotes the new image $I^{\prime }$ obtained by rotating the image I by angle θ. Additionally, cropping helps the model focus on important information in the image by randomly cropping parts of the image to increase the diversity of training samples:

(13) $$I^{\prime }=\mathrm{Crop}\left(I,\left(x_{1},y_{1}\right),\left(x_{2},y_{2}\right)\right),$$

where $\left(x_{1},y_{1}\right)$ and $\left(x_{2},y_{2}\right)$ represent the top-left and bottom-right coordinates of the cropping area, respectively.

#### 3.2.2. Audio Data Preprocessing and Augmentation

Audio data preprocessing and augmentation are also key steps in improving the model’s robustness and performance. Audio signals typically consist of a series of time-sequence data, and it may be difficult to extract meaningful features by directly processing these raw data. Therefore, several preprocessing operations are necessary. Common audio preprocessing methods include silence removal, speech rate normalization, and pitch enhancement. Silence removal is an important step in audio preprocessing, aiming to remove silent sections from the audio signal. Silent sections in audio can occupy storage space and potentially impact the model’s training efficiency. Therefore, by detecting silent portions and removing them, the quality of audio data can be effectively improved. Silence removal is typically performed by setting a threshold α to determine if the audio signal is silent:

(14) Silence(X,α)={t∣|X(t)|<α},

where X(t) represents the amplitude of the audio signal at time *t*, α is the silence threshold, and Silence(X,α) denotes the audio segments that are silent. By removing silent sections, ineffective information in the audio can be eliminated. Speech rate normalization refers to adjusting the speech rate of audio to a certain range so that speech inputs at different rates can be uniformly processed. Speech rate normalization is typically achieved by stretching or compressing the duration of the audio signal. Assuming the duration of the audio signal is *T* and the target duration after speech rate normalization is $T^{\prime }$, audio resampling can be performed by the following formula:

(15) $$X^{\prime }\left(t\right)=X\left(\frac{t}{T}\cdot T^{\prime }\right),$$

where $X^{\prime }\left(t\right)$ represents the normalized audio signal, *T* is the original duration, and $T^{\prime }$ is the target normalized duration. Speech rate normalization eliminates the impact of varying speech rates, ensuring consistency in the audio data. Pitch enhancement is a method used to change the pitch of the speech by adjusting its frequency, enabling the model to better adapt to varying speech characteristics. Pitch enhancement is typically achieved by modifying the frequency components of the audio signal, either stretching or compressing the frequency. Let F(X) represent the frequency components of the audio, and pitch enhancement can be represented as:

(16) $$F^{\prime }\left(X\right)=\alpha F\left(X\right),$$

where α is the pitch adjustment factor, F(X) represents the frequency components of the original audio signal, and $F^{\prime }\left(X\right)$ represents the frequency components after pitch enhancement. By enhancing the pitch, the pitch of the audio signal is adjusted, helping the model adapt to more variations in speech inputs. Spectral simulation extension is an important method in audio data augmentation. By performing spectral simulation extension on the original audio signal, diverse audio samples can be generated to enhance the model’s robustness. Spectral simulation usually involves randomly transforming the audio’s spectrogram, such as perturbing or scaling the magnitude of the spectrogram, thereby generating new audio samples. This approach allows the model to adapt to more complex and diverse audio inputs, improving its performance in real-world applications.

### 3.3. Proposed Method

**Figure 2:**
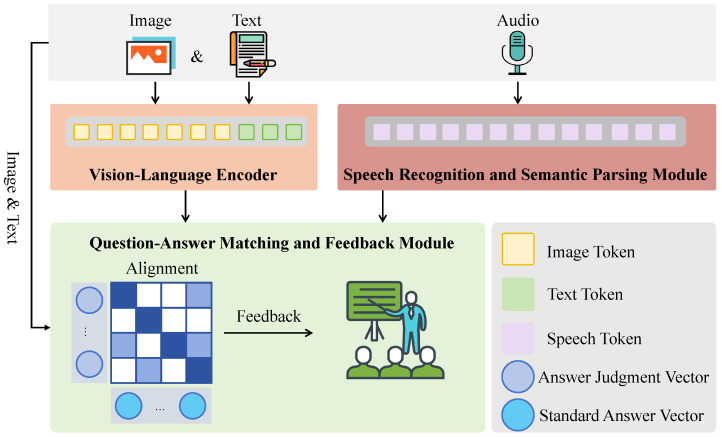
Overall architecture of the proposed cross-modal legal English question-answering system. The system sequentially integrates a visual-language encoding module, a speech recognition and semantic parsing module, and a question–answer matching and feedback module.

The proposed cross-modal legal English question-answering system is designed with the primary methodological objective of enabling accurate semantic alignment and interactive reasoning across visual, textual, and auditory modalities in legal educational contexts. Specifically, the system aims to (1) bridge the gap between perceptual evidence and legal language expression, (2) enhance the learner’s ability to comprehend and respond to complex multimodal case scenarios, and (3) provide interpretable, adaptive feedback to support progressive language acquisition. To achieve these goals, the architecture is composed of three sequential modules: a visual-language encoding module, a speech recognition and semantic parsing module, and a question–answer matching and feedback module. The processed case images and textual questions are first input into the visual-language encoding module, where deep feature extraction and normalization are performed to generate a unified visual-language semantic representation. Learners’ speech, collected via acoustic sensors, is then input into the speech recognition and semantic parsing module to be transcribed into text and to extract key information, which is subsequently aligned bidirectionally with the case semantics. Finally, in the question–answer matching and feedback module, similarity modeling and matching scoring are conducted based on multimodal fusion vectors, generating detailed feedback to assist language learning optimization, as shown in Figure 2. Additionally, in order to provide a clearer understanding of the system’s architecture and each component’s role, Table 2 summarizes the key modules, associated input modalities, underlying models, primary functions, and output formats.

#### 3.3.1. Visual-Language Encoding Module

**Figure 3:**
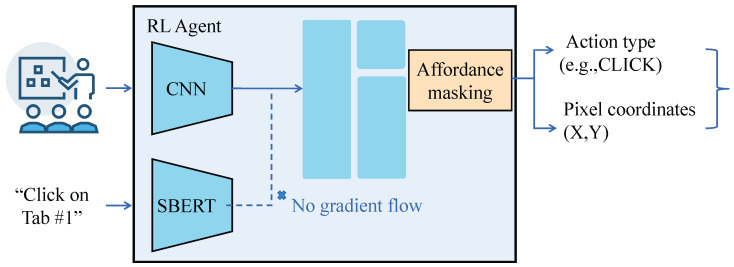
Illustration of the visual-language encoding module. The module extracts regional image features using a CNN and encodes textual semantics with a Sentence-BERT (SBERT) model to achieve deep multimodal correlation.

The visual-language encoding module is designed to achieve joint semantic understanding of case images and legal question texts, thereby generating a unified representation that serves as the foundation for subsequent cross-modal question answering. As illustrated in Figure 3, the input image is first processed through a convolutional backbone network to extract hierarchical spatial features. These features are subsequently segmented into patch tokens and embedded via a Transformer Encoder to capture global contextual relationships. In parallel, the legal question text is encoded using the Sentence-BERT model to obtain a sentence-level semantic vector that preserves the logical structure and linguistic nuances of legal discourse. To bridge the modality gap, visual and textual embeddings are projected into a shared semantic space, where cross-modal alignment is performed using a weighted attention mechanism. This process enables the integration of evidentiary visual cues with legal language semantics, resulting in a compact and interpretable multimodal feature representation. Specifically, the ResNet-50 architecture is employed as the backbone network in the image branch. ResNet-50 was chosen due to its strong balance between depth and computational efficiency, as well as its proven performance in capturing hierarchical features from structured and high-detail legal evidence images. Input case images are resized to 224×224 resolution and passed through four convolutional blocks, each containing a convolutional layer (with kernel size 3×3), batch normalization (BatchNorm), and ReLU activation units, progressively expanding the feature dimensions from 64 to 2048. After generating the convolutional feature maps, a multi-head self-attention mechanism (Multi-Head Attention) is introduced to meet the high-level semantic modeling requirements of cross-modal tasks. Each image is partitioned into 14×14 patch tokens and encoded using a Transformer Encoder, where each token has a dimensionality of D=512, with a depth of 6 layers and 8 attention heads per layer.

For the text branch, Sentence-BERT (SBERT) is utilized as the encoder, producing a D=768 dimensional semantic vector for each legal question text via a pretrained Transformer network, as it is specifically designed to generate high-quality sentence-level embeddings while maintaining contextual semantic coherence—an essential property for capturing the intricate logical structures and multi-clause dependencies commonly found in legal discourse. To mitigate the dimensionality discrepancy between modalities and facilitate subsequent fusion, the text vector is projected to 512 dimensions via a linear transformation, formulated as $t^{\prime }=W_{t}t+b_{t}$, where $W_{t}\in R^{512\times 768}$ is a learnable weight matrix and $b_{t}$ is the bias term. Visual and textual features are matched and aligned through a feature fusion layer. A weighted alignment strategy is adopted, whereby similarity scores $s_{i}=\cos\left(v_{i},t^{\prime }\right)$ are computed between each image token sequence $v_{1},v_{2},\ldots ,v_{n}$ and the projected text vector $t^{\prime }$, followed by Softmax normalization to obtain a weight distribution $p_{i}=\frac{\exp(s_{i})}{\sum_{j}\exp\left(s_{j}\right)}$. The fused visual semantic representation is calculated as a weighted sum $vfused=\sum {i=1}^{n}p_{i}v_{i}$, where n=196 denotes the number of image tokens. The final visual-language joint representation is obtained by concatenating the fused visual semantics and the text semantics, expressed as $z=[v_{fused};t^{\prime }]$, resulting in a 1024-dimensional unified encoded vector. To further enhance the module’s focus on key information regions, an affordance masking mechanism is introduced at the image feature extraction stage. The affordance masking mechanism prioritizes evidentiary image regions directly relevant to the current legal question, improving semantic integrity during fusion. Specifically, based on the content of the current question text, a mask matrix $M\in {0,1}^{14\times 14}$ is predicted to identify potential attention regions, retaining only the features where the mask value is 1 for subsequent processing. The mask prediction is achieved through a lightweight convolutional network, conditioned on the question text encoding vector. This mechanism effectively suppresses irrelevant background noise and non-target objects within the case images, thereby enabling the model to concentrate more accurately on evidential and reasoning-related visual clues. During training, the optimization objective of the visual-language encoding module is to maximize the similarity between positive image–text pairs (I,Q) while minimizing the similarity for negative pairs $\left(I,Q^{\prime }\right)$ or $\left(I^{\prime },Q\right)$. The loss function is based on an InfoNCE contrastive learning loss, defined as:

(17) $$Lcontrastive=-\log\frac{\exp(\cos\left(z_{I},z_{Q}\right)/\tau )}{\sum z^{\prime }\in Z\exp\left(\cos\left(z_{I},z^{\prime }\right)/\tau \right)},$$

where $z_{I}$ and $z_{Q}$ represent the encoded vectors for the image and text inputs respectively, τ denotes the temperature parameter, and Z refers to the set of all text encodings within the current batch. Through contrastive learning optimization, the model effectively brings correct image–text pairs closer in the shared semantic space while pushing apart mismatched pairs, thereby enhancing the cross-modal alignment capability of the encoding module. The design of the visual-language encoding module provides significant advantages for the targeted task. The combination of convolutional features and Transformer encoding enables the model to capture both local detail sensitivity and global contextual reasoning ability, allowing accurate association of critical case information with the corresponding legal questions. The affordance masking mechanism further strengthens the module’s focus on relevant regions, substantially improving the generalization performance across diverse legal case images. Moreover, the unified dimensionality design of the cross-modal encoding simplifies subsequent semantic matching operations and reduces inference latency, ensuring the responsiveness and interactivity of the system. Mathematically, the weighted feature fusion and contrastive learning training strategy allow the visual-language encoding module to form a more compact matching distribution in the feature space, thereby improving the matching accuracy and robustness under complex legal scenarios.

#### 3.3.2. Speech Recognition and Semantic Parsing Module

**Figure 4:**
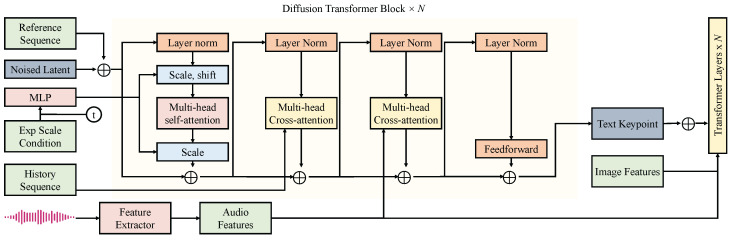
Illustration of the speech recognition and semantic parsing module. The module initially transcribes speech signals into audio features using an ASR model, incorporating inputs from case images, body keypoints, and hand coefficients to extract multisource features.

The speech recognition and semantic parsing module was designed to accurately extract textual information from learners’ speech input and further capture legal-related semantic features, thereby providing high-quality semantic representations for subsequent question–answer matching and feedback modules. As shown in Figure 4, the raw audio signals collected by acoustic sensors are first subjected to preprocessing steps including noise filtering, silence removal, and time–frequency feature extraction. These extracted features are then fed into a transformer-based automatic speech recognition (ASR) model to generate preliminary textual transcriptions. The ASR model was constructed based on a Transformer architecture composed of N=12 stacked Transformer Encoder layers, each consisting of a multi-head self-attention mechanism, a feedforward network, and a layer normalization module. The input features were initially downsampled via convolutional layers, where the raw audio feature dimensions were (T=160,F=80,C=1), with *T* representing time steps, *F* denoting frequency channels, and *C* corresponding to single-channel grayscale signals. After three convolutional blocks, each with a 3×3 kernel size and a 2×2 stride, the channel dimensions were progressively expanded to 32, 64, and 128, with the final output feature size reduced to (T/8,F/8,128).

The convolutional features were then flattened and augmented with positional encoding before being input into the Transformer Encoder. Each self-attention layer utilized H=8 attention heads, with each head having key, query, and value vector dimensions of $d_{k}=d_{q}=d_{v}=64$ and a feedforward layer with $d_{ff}=2048$ hidden units and a GELU activation function. The Transformer Encoder outputted a textual feature sequence $S=s_{1},s_{2},\ldots ,s_{n}$, where $s_{i}\in R^{512}$ represents the textual feature vector at the *i*-th time step. Due to the inherent limitations of conventional ASR architectures in capturing long-range semantic dependencies and maintaining contextual coherence in complex legal discourse, a series of extended cross-modal diffusion Transformer modules were introduced following the ASR model. These modules employ a multi-step semantic reconstruction mechanism, conditioned on historical semantic trajectories, to dynamically integrate contextual information while preserving the original speech features. This design enhances the model’s capacity to identify implicit logical structures and key legal terminology within extended spoken statements. The diffusion Transformer comprised N=8 diffusion blocks, each containing a multi-head cross-attention module, a multi-head self-attention module, a feedforward network, and a layer normalization module. The cross-modal attention utilized the current frame’s textual features as queries, with historical reference sequences serving as keys and values, thereby guiding the dynamic incorporation of historical semantic contexts into the current understanding process. After each cross-modal interaction, residual connections preserved the original semantic trajectory, while adaptive scale and shift operations adjusted feature magnitudes to ensure semantic stability during diffusion. To address feature consistency across different scales, a consistency fusion mechanism was introduced. After each block’s output, feature normalization and rescaling were performed, followed by minimizing the discrepancy with the initial reference features. The consistency fusion loss $L_{consistency}$ was defined as:

(18) $$Lconsistency=\frac{1}{n}\sum {i=1}^{n}{\left|s_{i}-r_{i}\right|}_{2}^{2},$$

where $s_{i}$ denotes the *i*-th output textual feature, $r_{i}$ represents the corresponding historical reference feature, and *n* is the total number of time steps. The consistency fusion loss ensures that semantic drift is minimized during multi-step reasoning, thereby preserving the core legal meanings in speech responses. Moreover, during multimodal joint reasoning involving visual evidence and textual answers, the semantic parsing module was aligned with the outputs of the visual-language encoding module. A cross-modal alignment loss $L_{align}$ was introduced, defined as:

(19) $$Lalign=-\frac{1}{n}\sum {i=1}^{n}\log\frac{\exp(\cos\left(s_{i},v_{i}\right)/\tau )}{\sum_{j=1}^{n}\exp\left(\cos\left(s_{i},v_{j}\right)/\tau \right)},$$

where $s_{i}$ denotes the parsed textual feature at time step *i*, $v_{i}$ represents the corresponding encoded vector of the case image–text pair, τ is the temperature parameter, and cos(·,·) indicates cosine similarity. By maximizing the similarity between correct text–image pairs and suppressing incorrect ones, the module enhanced feature discriminability during multimodal information fusion, thereby improving the precision of subsequent question–answer matching. The total loss function $L_{total}$ for this module was formulated as a weighted sum of the individual losses:

(20) $$Ltotal=\lambda_{1}LASR+\lambda_{2}Lconsistency+\lambda_{3}Lalign,$$

where $L_{ASR}$ is the standard cross-entropy loss for speech recognition and $\lambda_{1},\lambda_{2},\lambda_{3}$ are weighting coefficients set to 0.6,0.2,0.2, respectively, to achieve an optimal balance between recognition accuracy and cross-modal consistency. Through the above module design, the system ensured high accuracy in speech recognition while further enhancing the quality of the extracted legal semantic structures. By deeply collaborating with the visual-language encoding module, efficient alignment between case facts and language expression in the cross-modal legal English question-answering scenario was realized. Particularly, the integration of the diffusion Transformer and consistency fusion strategies significantly improved the system’s robustness and adaptability to complex inputs, such as accent variations, speech rate fluctuations, and case diversity, thereby ensuring broad applicability and high performance in practical educational settings.

#### 3.3.3. Question–Answer Matching and Feedback Module

**Figure 5:**
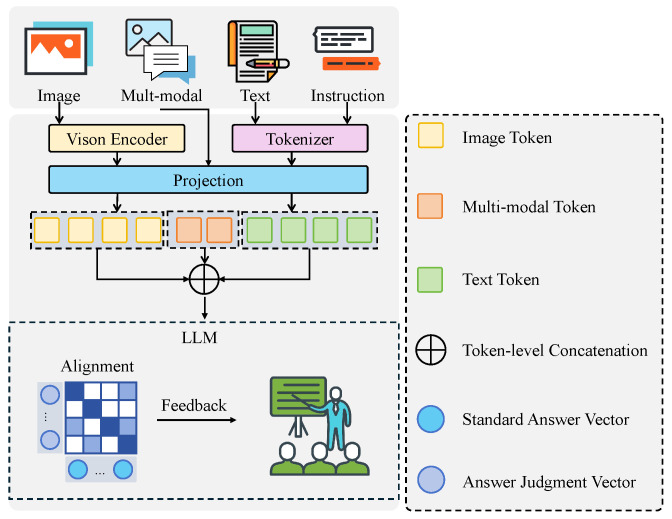
Illustration of the question–answer matching and feedback module. The module aims to compute the semantic alignment between learner responses and reference answers by integrating multimodal inputs, including case images, legal questions, spoken responses, and instructional prompts. Case images are processed via a Vision Transformer Encoder, while textual and speech features are embedded into a shared space through a projection mechanism. The resulting fused vector is fed into a large language model for contextual inference. A normalized similarity score is then calculated against the reference vector, guiding the generation of interpretable, template-based feedback informed by attention activations.

The question–answer matching and feedback module was designed to achieve deep semantic alignment between learners’ spoken responses, case images, and legal question texts, while generating personalized and targeted feedback to enhance the interactivity and instructional effectiveness of the cross-modal legal English question-answering system. As shown in Figure 5, the module comprises a vision encoder, a projection layer, a large language model (LLM), and a normalization and inference layer. Various data sources, including images, multimodal fused features (outputs from the visual-language encoding and speech parsing modules), textual questions, and task instructions, were preprocessed and embedded through specific paths before being fused, matched, and output.

The input components included case images, multimodal fusion features, textual question content, and prompt instructions. Case images were processed through a Vision Encoder based on the ViT-B/16 (Vision Transformer Base) architecture, which was selected for its superior capability in modeling long-range dependencies and global context—essential for capturing complex evidentiary relationships in legal imagery. This architecture consists of 12 transformer blocks with a hidden width of 768 and 12 self-attention heads, each with a dimension of 64, maintaining an overall feature dimension of 768. Images were partitioned into 16×16 patches, each linearly projected into a 768-dimensional vector and then passed through the Transformer to model global relationships. After encoding, a visual semantic vector $v\in R^{768}$ was produced. Correspondingly, textual content and instructions were tokenized and embedded into 768-dimensional vectors, ensuring feature space consistency with the visual vectors. To effectively integrate image, text, and other modality inputs within a unified feature space, a projection layer was introduced after the Vision Encoder, defined by the linear mapping:

(21) $$v^{\prime }=W_{p}v+b_{p},$$

where $W_{p}\in R^{768\times 768}$ and $b_{p}$ is a learnable bias term. This projection operation retained the feature dimension while adjusting the distribution of visual features to better align with the input requirements of the large language model. After projection, all modality inputs were concatenated within the embedding space to form a complete multimodal input sequence, serving as the basis for subsequent reasoning. The multimodal input sequence was fed into a pretrained large language model based on the OPT-1.3B architecture, which was selected as the backbone after comparative evaluation of multiple open-source models of varying parameter scales, as it provides a favorable balance between inference capability and computational efficiency—consisting of 24 Transformer blocks, each with a width of 2048 and 32 self-attention heads, each head with a dimension of 64. Through layer normalization and multi-head attention mechanisms, the model implicitly learned cross-modal relationships during inference. The large language model, via context modeling, established high-level semantic correspondences between case images, question texts, and learners’ responses and outputted a final answer judgment vector $o\in R^{2048}$. To evaluate the matching degree between learners’ answers and standard answers, a normalization and matching layer was introduced. The matching process employed an inner product similarity metric, where the output vectors were normalized and the cosine similarity with the standard answer vector $o_{std}$ was computed as follows:

(22) $$\mathrm{sim}\left(o,ostd\right)=\frac{o\cdot ostd}{\left|o|\right|o_{std}|}.$$

The matching score was then input into the feedback generation submodule. Based on the score and local attention activations, personalized feedback texts were generated following a template-based strategy. To further enhance interpretability, the feedback module integrates an attention visualization mechanism and modular template design to support the traceability of decision paths. Learners can review which visual or linguistic components were emphasized during answer generation, increasing the transparency of the assessment and enabling more targeted improvement. Although the system is built upon predefined structures, the feedback content is not strictly fixed. Instead, it dynamically selects and assembles appropriate prompt phrases from a template pool based on the question–answer matching score and attention-weighted focus regions, embedding specific fragments from the learner’s response to produce targeted and context-aware feedback. Specifically, when the similarity exceeded the threshold θ=0.85, positive feedback was provided, encouraging learners to attempt more complex reasoning. When the similarity fell within the range 0.6<sim<0.85, the system activated partially correct templates and, by identifying missing or misaligned elements through attention distribution, offered interpretable suggestions for refinement. In cases where the similarity dropped below 0.6 and the learner’s response deviated significantly from expectations, the system prompted a re-evaluation of key case elements by presenting scaffolded instructional cues, including decomposed sub-questions and reflective prompts, thereby enhancing the openness and guidance of the feedback mechanism. To improve feedback consistency and contextual coherence across multiple rounds of interaction, a cumulative feedback loss $L_{feedback}$ was designed, defined as:

(23) $$Lfeedback=\frac{1}{N}\sum {i=1}^{N}{\left(1-\mathrm{sim}\left(oi,ostd,i\right)\right)}^{2},$$

where *N* represents the number of historical interaction rounds, oi is the output vector at the *i*-th round, and ostd,i is the corresponding standard answer vector. This loss encouraged the model to maintain output consistency across interactions, avoiding feedback fluctuations caused by local feature noise, thereby providing learners with a stable and coherent learning experience. The design of this module offers substantial advantages for the targeted task. On one hand, by introducing the Vision Encoder and projection mechanism, deep integration of case image information and textual semantics within the feature space was achieved, requiring learners to not only comprehend text but also accurately reason based on visual evidence. On the other hand, by leveraging the context modeling capabilities of the large language model, complex implicit cross-modal correspondences within case reasoning processes were effectively captured, enhancing the model’s understanding of complex responses. Furthermore, the introduction of the normalization-based matching strategy and cumulative feedback loss ensured the precision of answer matching and the stability of feedback generation, significantly enhancing learners’ comprehensive application abilities and learning outcomes in authentic legal English scenarios.

## 4. Results and Discussion

### 4.1. Hardware and Software

The hardware platform of this system includes a high-performance workstation equipped with the latest NVIDIA A100 GPU (Nvidia, Santa Clara, CA, USA), which provides powerful computing capabilities and can efficiently support deep learning model training and inference. The workstation also features 32GB of memory and a 1TB solid-state drive, providing ample storage and processing space to handle large-scale dataset loading and real-time processing requirements. For input devices, we use a high-definition camera and a high-sensitivity microphone, with the former capturing high-quality legal case images and the latter accurately capturing a learner’s speech input. Additionally, the system is equipped with multiple external hard drives for data backup and storage, ensuring data security and the efficient operation of the system. In terms of software, the system is developed in a Linux-based environment, specifically using Ubuntu 20.04 LTS to ensure stability and compatibility. The deep learning framework used is primarily PyTorch 2.1, with TensorFlow 2.14 utilized for the optimization and training of specific tasks. To support multimodal input processing and fusion, the system relies on the pretrained model library provided by Hugging Face, particularly models like BERT and CLIP for multimodal deep learning. Data preprocessing and augmentation are handled using open-source libraries such as OpenCV and librosa, with NumPy and Pandas used for data processing and management. During development, Docker 4.28 is employed for containerized deployment, ensuring environment portability and scalability. With this careful configuration of both hardware and software, the system can efficiently process large-scale data and perform complex deep learning tasks, ensuring stability and high efficiency in real-world applications.

### 4.2. Hyperparameters

In this experiment, the dataset is split into training, validation, and test sets with a standard ratio, where 70% of the data is used for training, 15% for validation, and 15% for testing. This division ensures that sufficient training data are available for model learning while the validation and test sets effectively assess the model’s generalization ability. Regarding hyperparameter settings, several key hyperparameters of the model were adjusted and optimized to ensure optimal performance across different tasks. The learning rate α is set to 0.0001, selected through a grid search method from several candidate values. The batch size $batch_{s}ize$ is set to 32 to balance training efficiency and model performance. The Adam optimizer is used for training the deep learning models, with $\beta_{1}$ and $\beta_{2}$ set to 0.9 and 0.999, respectively, to ensure stable gradient updates. During the model training process, a 5-fold cross-validation strategy is employed to train and validate the model multiple times, avoiding overfitting and improving the model’s stability and reliability. The results of cross-validation help further adjust the model’s hyperparameters, optimizing its performance across different datasets. Through this rigorous hyperparameter tuning and cross-validation process, the system’s performance in practical tasks is maximized.

### 4.3. Evaluation Metrics

In the evaluation of question-answering accuracy, common metrics include BLEU, ROUGE, Precision, Recall, and Accuracy, which help measure the performance of the system on a legal question set. First, BLEU is used to measure the n-gram matching between the generated answer and the reference answer, while ROUGE evaluates the recall between the generated text and the reference text. Precision calculates the proportion of correct answers generated by the system, Recall measures the proportion of correct answers that the system can identify, and Accuracy provides an overall measure of the correctness of all predictions. Their mathematical formulas are as follows:

(24) $$\mathrm{BLEU}\left(n\right)=\min\left(1,\frac{p_{n}\left(S\right)}{\max(p_{n}\left(C\right))}\right)$$

(25) $$\mathrm{ROUGE}\text{-}L=\frac{LCS(S,C)}{\mathrm{len}(C)}$$

(26) $$\mathrm{Precision}=\frac{\mathrm{TP}}{\mathrm{TP}+\mathrm{FP}}$$

(27) $$\mathrm{Recall}=\frac{\mathrm{TP}}{\mathrm{TP}+\mathrm{FN}}$$

(28) $$\mathrm{Accuracy}=\frac{\mathrm{TP}+\mathrm{TN}}{\mathrm{TP}+\mathrm{TN}+\mathrm{FP}+\mathrm{FN}},$$

where TP represents the number of correct answers identified by the system, FP represents the number of incorrectly identified answers, FN represents the number of missed correct answers, TN represents the number of correctly identified non-answers, LCS(S,C) represents the longest common subsequence length between the generated answer S and the reference answer C, and len(C) is the length of the reference answer.

In addition to system-level accuracy, three user-centered metrics are introduced to evaluate learning improvement and subjective experience: Understanding Improvement, Expression Improvement, and Satisfaction Score. These metrics are designed to quantify the system’s impact on users’ comprehension, expressive ability, and satisfaction in the context of legal English learning. Understanding Improvement measures the learner’s progress in understanding legal scenarios and factual content before and after using the system. Expression Improvement reflects the extent to which learners enhance their legal English output, including grammatical correctness, use of terminology, and logical coherence. Satisfaction Score captures users’ perceived experience with the system in terms of usability, feedback quality, and instructional value. The corresponding formulas for these metrics are defined as follows:

(29) $$\mathrm{Understanding}\;\mathrm{Improvement}=\frac{S_{\mathrm{post}}-S_{\mathrm{pre}}}{S_{\mathrm{pre}}}$$

(30) $$\mathrm{Expression}\;\mathrm{Improvement}=\frac{E_{\mathrm{post}}-E_{\mathrm{pre}}}{E_{\mathrm{pre}}}$$

(31) $$\mathrm{Satisfaction}\;\mathrm{Score}=\frac{1}{N}\sum_{i=1}^{N}s_{i}$$

where $S_{\mathrm{pre}}$ and $S_{\mathrm{post}}$ represent the learner’s average pre-test and post-test scores on case comprehension tasks, respectively. $E_{\mathrm{pre}}$ and $E_{\mathrm{post}}$ denote the average scores for expressive legal tasks before and after system interaction. $s_{i}$ is the subjective satisfaction rating provided by the *i*-th participant on a five-point Likert scale and *N* is the total number of participants.

### 4.4. Baseline

In this experiment, the baseline models include three commonly used systems: the traditional ASR+SVM classifier [52], unimodal BERT question-answering system [53], and image-retrieval-based question-answering model [54]. These models are meticulously selected to represent the typical paradigms of unimodal or weakly cross-modal question-answering systems. Specifically, the ASR+SVM classifier reflects a traditional pipeline approach based on speech-driven processing, while the unimodal BERT QA system exemplifies text-driven semantic reasoning. The image-retrieval-based QA model, on the other hand, serves as a representative of vision-driven query methods. These baselines were chosen to provide a comparative framework for evaluating the proposed method’s performance gains in multimodal semantic alignment, deep cross-modal reasoning, and interactive feedback. By benchmarking against these classical systems, it becomes possible to clearly demonstrate the practical advantages of incorporating advanced multimodal fusion and cross-modal attention mechanisms. The observed improvements thus not only validate the effectiveness of the proposed framework but also highlight the limitations of shallow or unimodal strategies in complex legal education scenarios that require nuanced semantic understanding and evidentiary reasoning across modalities.

### 4.5. Experimental Results of Question-Answering Models

This experiment was designed to evaluate the overall performance of different question-answering models in the context of cross-modal legal English tasks, aiming to verify the system’s capability to generate accurate responses after understanding complex case images and textual questions. The objective of the experiment was to compare the proposed method with traditional baseline methods, thereby validating the effectiveness of introducing multimodal fusion, visual-guided mechanisms, and deep semantic alignment strategies in improving answer accuracy, text generation quality, and overall reasoning ability. Five evaluation metrics, namely BLEU, ROUGE, Precision, Recall, and Accuracy, were utilized to comprehensively assess the performance, covering content generation quality, retrieval coverage, and classification accuracy, thereby fully reflecting the system’s practical application potential in authentic legal education scenarios involving complex multimodal inputs.

As shown in Table 3, the Traditional ASR+SVM Classifier exhibited the weakest performance, primarily due to its separation of speech recognition and question–answer judgment processes, which failed to establish deep semantic connections between images and texts, resulting in lower BLEU and ROUGE scores. The image-retrieval-based QA model showed moderate improvement; however, its reliance on direct feature matching without text generation capability led to limitations in Precision and Recall. The unimodal BERT QA system achieved better generation quality through deep textual understanding, yet its lack of visual evidence incorporation resulted in incomplete comprehension when facing cross-modal problems. In contrast, the proposed method employed a three-modality deep encoding of vision, language, and speech and introduced a cross-modal attention mechanism to achieve high-level alignment among case images, question texts, and user responses, thus obtaining the best performance across all evaluation metrics. Mathematically, by utilizing contrastive learning and consistency optimization strategies, the proposed method enabled different modalities to form a more compact distribution within a unified feature space, significantly improving the modeling and reasoning ability for complex semantic structures.

### 4.6. Experimental Results of Multimodal Matching Models

This experiment aimed to evaluate the performance of different cross-modal models in the image–text alignment task, validating the effectiveness of the proposed unified encoding framework that fuses visual evidence, textual questions, and spoken responses. The objective of the experiment was to compare the performance of mainstream cross-modal baseline models, including VisualBERT, CLIP, World2Vec, SpeechBERT, LXMERT, ALBEF, and BLIP, analyzing their strengths and weaknesses in multimodal feature extraction, alignment modeling, and retrieval reasoning, thereby demonstrating the proposed method’s adaptability and improvement in handling complex legal scene alignments. Evaluation metrics included Matching Accuracy, Recall@1, Recall@5, and Mean Reciprocal Rank (MRR), comprehensively reflecting matching ability and reasoning depth from four perspectives: overall pairing accuracy, top-1 retrieval hit rate, multi-result retrieval coverage, and average ranking quality.

As shown in Table 4, VisualBERT exhibited the weakest performance across all metrics, mainly because its early-stage feature concatenation approach failed to effectively model fine-grained alignments between image regions and text segments. LXMERT introduced a separated encoder and cross-modal interaction mechanism, achieving slight improvement over VisualBERT; however, its single-step feature fusion strategy remained inadequate for complex image–text reasoning scenarios. Word2Vec showed marginal improvements in overall accuracy compared to VisualBERT, but due to its reliance on static, context-independent word embeddings, it lacked the representational capacity to capture semantic nuance in legal discourse or align with high-level visual concepts, resulting in limited Recall and MRR. SpeechBERT, leveraging speech–text joint modeling, achieved better cross-modal alignment by integrating BERT’s semantic reasoning capabilities with audio inputs. However, the architecture’s heavy dependence on speech signal quality and lack of visual grounding limited its robustness in tasks requiring multi-source evidentiary reasoning. CLIP demonstrated notable performance gains by leveraging contrastive learning with large-scale pretraining, thereby constructing a robust vision-language embedding space. Nonetheless, due to the absence of targeted fine-tuning, its effectiveness in handling nuanced legal evidence was limited, particularly in low-resource, high-reasoning scenarios. ALBEF further enhanced cross-modal representation by employing a vision–language pretraining strategy that integrated image–text matching, image–text contrastive learning, and masked language modeling in a unified framework. This design enabled better alignment and contextual understanding between modalities. However, ALBEF still relied on pre-extracted image features, which constrained its adaptability to domain-specific visual semantics in legal scenes. BLIP, as a more recent advancement, combined vision–language pretraining with a captioning-guided bootstrapping strategy, facilitating end-to-end learning of both unimodal and multimodal representations. Its architecture allowed for stronger text generation capabilities and more context-aware alignment. Consequently, BLIP outperformed previous baselines across all metrics and demonstrated robust reasoning ability in multimodal scenarios. The proposed method, through the fusion modeling of vision, text, and speech modalities and the introduction of dynamic attention and multi-level alignment optimization strategies, achieved deeper unification from the feature level to the semantic level. Different modalities were more compactly and discriminatively embedded in the feature space, leading to the best performance in Matching Accuracy, Recall@1, Recall@5, and MRR metrics. These results validated the effectiveness and superiority of the proposed mathematical mechanisms in cross-modal legal English question-answering scenarios.

### 4.7. Experimental Results of User Study on Learning Improvement

A total of N=60 participants were recruited for the user study, consisting of senior undergraduate and graduate students enrolled in legal English courses at three universities. Based on standardized English placement test results, participants were balanced by gender and language proficiency and then randomly assigned to one of four instructional groups, corresponding to the following teaching modes: traditional textbook-based instruction, conventional oral practice systems, a unimodal question-answering system, and the proposed multimodal question-answering system. Each group included 15 participants. Data collection was conducted over a four-week instructional period under a unified teaching schedule. Structured comprehension and expression assessments were administered both before and after instruction to measure learning progress. All participants completed case-based legal expression tasks of equivalent quantity and difficulty under instructor supervision. Scoring was performed independently by two legal education experts following the Common European Framework of Reference for Languages (CEFR) standards. Inter-rater reliability was validated using Cohen’s κ=0.87, indicating a high level of agreement. Learning outcomes were quantitatively evaluated across three dimensions: improvement in comprehension, improvement in legal expression, and user satisfaction. The first two dimensions were calculated as the difference between pre-test and post-test performance, while satisfaction scores were collected using a standardized five-point Likert scale. In addition, participants’ open-ended feedback was systematically coded and analyzed using thematic analysis to identify recurring concerns regarding system functionality, interactivity, and perceived instructional value. For statistical analysis, repeated measures analysis of variance (ANOVA) was employed to assess both within-group learning gains and between-group differences. By systematically comparing the proposed method against traditional textbook instruction, oral practice systems, and unimodal QA models, the experiment comprehensively demonstrated the effectiveness of the proposed approach in enhancing multimodal reasoning capabilities, improving legal expression accuracy, and fostering learner motivation. This study was conducted through authentic classroom interventions to evaluate the impact of different instructional methods on learners’ comprehension and expression gains, with the aim of validating the practical applicability and pedagogical value of the proposed cross-modal legal English question-answering system.

As shown in Table 5, the traditional textbook-based teaching method exhibited the weakest performance across all metrics, indicating that relying solely on textual materials was insufficient to stimulate learners’ deep understanding of complex legal scenarios. Although the traditional oral practice system showed slight improvement in expression ability, the absence of multimodal evidence limited the overall enhancement of understanding. The unimodal question-answering system achieved moderate improvements in both understanding and expression by incorporating deep text comprehension mechanisms; however, the lack of visual evidence still resulted in an incomplete grasp of semantics. In contrast, the proposed multimodal question-answering system integrated visual, linguistic, and auditory information into a unified semantic space, guiding learners to reason and express based on multimodal evidence, thereby achieving significant advantages across understanding, expression, and satisfaction metrics. Mathematically, multimodal feature alignment and dynamic feedback mechanisms effectively enhanced learners’ cognitive load management and semantic transfer capabilities under complex input conditions, ensuring the system’s high applicability and robust performance in real educational environments.

### 4.8. Generalization Evaluation on Open Multimodal Dataset

To further verify the generalizability and robustness of the proposed method, we conducted additional experiments on the publicly available MultimodalQA dataset. This dataset features complex, multimodal legal question-answering tasks involving textual descriptions, visual references, and spoken queries. As presented in Table 6, the proposed method significantly outperformed baseline approaches across all five evaluation metrics: BLEU, ROUGE, Precision, Recall, and Accuracy.

Traditional ASR+SVM models demonstrated limited performance due to their modular pipeline and lack of deep semantic alignment. The image retrieval QA baseline showed slight improvements by incorporating visual cues but remained constrained by shallow retrieval logic. The unimodal BERT QA model achieved better performance by leveraging pretrained language understanding, yet it failed to effectively integrate cross-modal context. In contrast, the proposed method, which jointly encodes and aligns textual, visual, and auditory modalities within a unified framework, achieved superior performance across all metrics (BLEU: 0.83, ROUGE: 0.85, Precision: 0.87, Recall: 0.83, Accuracy: 0.86). These results demonstrate the method’s strong capacity for multimodal reasoning and reinforce its adaptability in complex, open-domain legal QA scenarios beyond the in-house dataset.

### 4.9. Ablation Study on Learners with Varying English Proficiency

To further examine the adaptability and effectiveness of the proposed system across user groups with diverse language abilities, we conducted a user study stratified by English proficiency levels. Participants were categorized into three groups, high-level, medium-level, and low-level learners, based on standardized language assessment scores. The evaluation focused on three key indicators: understanding improvement, expression improvement, and user satisfaction, as shown in Table 7.

The results demonstrate that all groups benefited from the system, with noticeable improvements in both comprehension and expressive skills. High-level learners showed the highest gains (understanding: 0.81, expression: 0.79), reflecting their greater ability to integrate multimodal cues and utilize feedback for refinement. Medium-level learners also exhibited consistent improvements (0.76 and 0.74), indicating that the system supports intermediate learners in bridging semantic gaps during multimodal interactions. Although the low-level group achieved relatively lower gains (0.72 and 0.69), their satisfaction score remained high (0.83), suggesting that the system’s interface and scaffolding mechanisms provided accessible and encouraging learning experiences, even for users with limited language proficiency. These findings indicate that the proposed method maintains stable usability and pedagogical effectiveness across a wide range of learner profiles, supporting its broader applicability in real-world legal English education settings.

### 4.10. Discussion

#### 4.10.1. Practical Implications and Performance Analysis

A cross-modal legal English question-answering system based on visual and acoustic sensor inputs was proposed to address the limitations of traditional legal English teaching, such as reliance on single-modality input, insufficient interactivity, and difficulties in semantic transfer. Through systematic experimental designs and multidimensional evaluation metrics, the proposed method was validated for its significant advantages in complex case comprehension, legal expression training, and enhancement of learning experiences. In the evaluation of question-answering accuracy, traditional ASR+SVM classifiers and retrieval-based QA models exhibited evident limitations when handling cross-modal inputs, particularly due to their lack of deep semantic reasoning and visual evidence modeling capabilities. Although the unimodal BERT system demonstrated improvements in textual understanding, its neglect of image and audio inputs resulted in insufficient reasoning depth. In contrast, the proposed method, through a fusion mechanism of vision, language, and speech modalities and the introduction of a cross-modal attention mechanism, significantly enhanced the system’s capability to capture complex relationships between case evidence and legal questions, thus achieving superior performance across BLEU, ROUGE, Precision, Recall, and Accuracy metrics. In the analysis of multimodal matching performance, the comparison among different models further highlighted the critical role of cross-modal fusion strategies in improving image–text alignment. Although VisualBERT and LXMERT initially achieved semantic mapping between images and text, their lack of fine-grained alignment and optimization led to notable deficiencies in matching accuracy and retrieval performance. CLIP, while establishing a stronger unified cross-modal space through large-scale contrastive learning, lacked fine-tuning for small-sample complex legal reasoning tasks, leading to performance losses on fine-grained evidence tasks. By introducing dynamic attention mechanisms and a unified encoding space design, the proposed method reinforced deep fine-grained cross-modal relationship modeling, effectively addressing challenges such as fragmented evidence and long reasoning chains in legal image–text scenarios. Consequently, the proposed method achieved the best performance across Matching Accuracy, Recall@1, Recall@5, and MRR metrics, demonstrating that shallow feature fusion is insufficient for understanding complex scenarios and deep multimodal fusion with structured reasoning is indispensable.

The results of the user study further confirmed that improvements in model performance translated into tangible enhancements in educational effectiveness. In real classroom environments, the proposed method significantly enhanced learners’ case comprehension depth and English legal expression accuracy, with satisfaction scores markedly surpassing those of traditional and unimodal teaching methods. Traditional textbook- or oral-based methods, due to a lack of contextual sensing and interactive reasoning, showed limited learning effectiveness, while unimodal QA systems improved some aspects of expression but failed to cultivate the systematic reasoning abilities necessary for complex evidence-based reasoning. Through unified reasoning supported by visual, linguistic, and auditory modalities, learners naturally constructed factual narratives and legal reasoning chains, demonstrating superior language transfer and critical thinking capabilities. These results reflect the practical value and application potential of intelligent multimodal perception and deep fusion technologies in legal English education, offering empirical validation and future references for the design of intelligent multimodal interactive systems in foreign language education.

#### 4.10.2. Methodological Reflections and Scientific Contributions

While the practical advantages of the proposed system have been substantiated through quantitative metrics and user studies, it is equally important to reflect on its methodological and scientific contributions within the broader context of multimodal research.

Recent research has increasingly emphasized the theoretical and empirical advantages of multimodal architectures over unimodal or bimodal counterparts. Multimodal learning enables richer semantic representation by leveraging complementary information across modalities, enhancing generalization in both perception and reasoning tasks [55]. For instance, studies in visual question answering and grounded language understanding have shown that combining vision and language modalities improves spatial inference and referential accuracy [56]. Similarly, integrating acoustic information has been found to reinforce temporal modeling and improve robustness to ambiguous textual or visual cues [57]. In educational contexts, multimodal approaches have demonstrated improvements in learner engagement, feedback personalization, and cognitive load management, particularly when integrated with adaptive feedback systems [58]. These findings suggest that multimodal systems are not merely additive in capability but fundamentally more expressive and cognitively aligned with human information processing.

This work integrates three distinct modalities—vision, language, and speech—into a unified representation space, extending prior efforts that typically focused on bimodal fusion [19,20]. The use of dynamic attention mechanisms, affordance-driven visual masking, and multi-level alignment losses contributes to a deeper, fine-grained modeling of heterogeneous legal inputs. These strategies address key challenges in multimodal learning, such as modality imbalance, semantic drift, and evidence grounding, which are often overlooked in earlier designs such as the VisualBERT [24] or wav2vec-only pipelines [25]. Furthermore, unlike traditional question-answering systems that adopt shallow concatenation or single-stage fusion, the proposed framework introduces structured semantic constraints (e.g., contrastive alignment loss, consistency fusion loss), enabling hierarchical alignment from the feature to semantic levels. This aligns with recent scientific perspectives emphasizing the need for “structured explainability” and transparent decision pathways in multimodal educational systems [18,21]. Compared to baseline models, the inclusion of a speech-informed semantic parsing module in conjunction with image-based evidence understanding represents a novel direction. Prior studies have separately validated the role of vision–text [59,60] and speech–text models [61], but the legal domain presents a unique challenge where factual accuracy, logical rigor, and multimodal alignment must co-exist—an area underexplored in the current research. In this context, the proposed integration strategy enhances the interpretability, adaptability, and semantic depth required for high-stakes, domain-specific applications.

In sum, this study bridges a critical gap in the literature by introducing a scientifically grounded, pedagogically oriented multimodal learning system tailored for legal education. It demonstrates that educational AI models benefit not only from larger datasets or more parameters, but from principled integration of cross-modal structure, interpretability mechanisms, and domain-aware representation strategies.

### 4.11. Limitations and Future Work

Despite the strong performance demonstrated by the proposed cross-modal legal English question-answering system based on visual and auditory sensor inputs in terms of question-answering accuracy, multimodal matching capability, and user learning outcomes, several limitations remain. At present, the system primarily focuses on three data types: case images, textual questions, and spoken responses. More complex legal document forms, such as judicial opinions, contractual clauses, and courtroom transcripts, have not yet been incorporated, which limits the model’s applicability in tasks with high semantic complexity. In addition, the current system remains primarily designed for English-language contexts, and its transferability and generalization ability in multilingual settings require further investigation. Although the principles of informed consent and anonymization were followed during data collection, ethical and privacy risks associated with image and speech sensor inputs remain a critical concern in real-world educational deployments. Future iterations of the system will integrate data desensitization mechanisms, differential privacy algorithms, and ethical review procedures to ensure compliance, transparency, and user controllability. In terms of functional expansion, subsequent research will further explore model compression and inference optimization strategies suitable for deployment on resource-constrained devices. Additional sensor modalities, such as tactile feedback, eye-tracking, and physiological signals, will be introduced to construct a more immersive and realistic language learning experience. On this basis, future studies will focus on the architectural exploration of next-generation cross-modal frameworks, including the integration of graph neural networks with multi-scale attention mechanisms, the construction of multimodal causal reasoning models, and the implementation of dynamic context modeling strategies. These methods are expected to enhance the model’s capacity to capture structured semantic relationships across heterogeneous data sources and to advance multimodal intelligent language learning systems toward stronger generalization and interactivity, thereby extending the theoretical boundaries of human–AI collaborative language education.

## 5. Conclusions

With the rapid development of artificial intelligence and sensor technologies, traditional legal English teaching models have shown increasing limitations in multimodal understanding, interactive reasoning, and language transfer capabilities. To address the challenges of relying solely on text-based input, a lack of semantic grounding in perceptual evidence, and falling short in real-world legal scenarios, this paper proposed a cross-modal legal English question-answering system based on visual and acoustic sensor inputs. The core innovation lies in the construction of a unified vision–language–speech fusion framework, enhanced by dynamic attention mechanisms and multimodal consistency optimization strategies. This design enables deep perception of complex legal cases and supports structured training in legal reasoning and English expression. Extensive experiments demonstrate that the proposed system significantly outperforms traditional baselines—such as ASR+SVM classifiers, image-retrieval-based models, and unimodal BERT systems—achieving an Accuracy of 0.87, a Precision of 0.88, and a Recall of 0.85. User studies further confirm its educational effectiveness, with learners’ comprehension, expressive ability, and satisfaction scores significantly improved in real teaching scenarios. From a theoretical perspective, this study contributes to advancing multimodal semantic learning in legal language education by integrating structured perception, language alignment, and feedback generation within an interactive learning framework. Moreover, the incorporation of semantic feedback and image–speech alignment mechanisms provides a path toward improving system interpretability and learner trust. Nonetheless, certain limitations remain. The current system focuses on a limited set of input modalities and domain-specific tasks, and its generalizability to more complex legal texts, multilingual environments, and cross-cultural scenarios remains to be validated. In addition, ethical and privacy issues surrounding sensor-based data collection call for stricter compliance frameworks in real deployments. Future research will explore lightweight model architectures suitable for resource-constrained environments, incorporate additional sensor modalities (e.g., haptic and gaze data), and integrate privacy-preserving mechanisms such as data anonymization and differential privacy. More importantly, new research directions will focus on the development of adaptable multimodal architectures, including graph-based reasoning networks, causal multimodal models, and dynamic context-aware learning frameworks, thereby pushing the boundaries of intelligent language education systems. In summary, this work not only presents a practical and effective solution to current challenges in legal English learning but also provides a robust theoretical and technical foundation for future advances in multimodal intelligent education systems.

## Figures and Tables

**Table 1:** Data quantity for different types of multimodal legal English resources.

Data Type	Quantity
Case Scene Images (CourtListener)	1247
Evidence Document Images (FindLaw)	1372
Legal Document Scans (Wikimedia Commons)	1098
Legal QA Text Pairs (Supreme Court/FindLaw/Harvard Law Review)	1520
Learner Response Audios (Recorded by Blue Yeti)	1214

**Table 2:** Overview of module functions in the proposed cross-modal legal QA system.

Module Name	Input Modalities	Core Models Used	Primary Function	Output Format
Visual-Language Encoding Module	Case image, question text	ResNet-50, SBERT, Multi-head Attention	Extracts deep visual and textual features and generates a unified semantic representation	1024-dim fused vector
Speech Recognition and Semantic Parsing	Learner’s spoken response	Transformer ASR, Diffusion Transformer	Converts audio to text and extracts legal semantic structure aligned with visual-question context	Sequence of 512-dim vectors
Question–Answer Matching and Feedback	Fused visual, text, and speech features	ViT-B/16, OPT-1.3B LLM, Feedback Generator	Performs multimodal answer matching and generates adaptive, interpretable feedback for learners	Matching score and feedback

**Table 3:** Experimental results of question-answering models.

Model	BLEU	ROUGE	Precision	Recall	Accuracy
Traditional ASR+SVM Classifier [52]	0.62	0.65	0.64	0.61	0.63
Image-Retrieval-based QA Model [54]	0.68	0.70	0.69	0.67	0.68
Unimodal BERT QA System [53]	0.74	0.77	0.76	0.74	0.75
Proposed Method	0.83	0.86	0.88	0.85	0.87

**Table 4:** Experimental results of multimodal matching models.

Model	Matching Accuracy	Recall@1	Recall@5	MRR
VisualBERT	0.71	0.69	0.77	0.73
CLIP	0.79	0.77	0.84	0.80
Word2Vec	0.72	0.71	0.78	0.75
SpeechBERT	0.77	0.75	0.82	0.80
LXMERT	0.74	0.72	0.80	0.76
ALBEF	0.80	0.78	0.84	0.82
BLIP	0.81	0.80	0.86	0.83
Proposed Method	0.85	0.83	0.90	0.87

**Table 5:** Experimental results of user study on learning improvement.

Method	Understanding Improvement	Expression Improvement	Satisfaction Score
Textbook-based Teaching	0.45	0.40	0.60
Traditional Oral Practice System	0.52	0.48	0.65
Unimodal QA System	0.63	0.58	0.72
Proposed Method	0.78	0.75	0.88

**Table 6:** Performance on Open Dataset Multimodal QA.

Model	BLEU	ROUGE	Precision	Recall	Accuracy
Traditional ASR+SVM	0.62	0.64	0.63	0.60	0.63
Image Retrieval QA	0.67	0.68	0.68	0.65	0.67
Unimodal BERT QA	0.73	0.76	0.75	0.73	0.73
**Proposed Method**	**0.83**	**0.85**	**0.87**	**0.83**	**0.86**

**Table 7:** User study on learners with different English proficiency levels.

Group	Understanding Improvement	Expression Improvement	Satisfaction Score
High-Level Learners	0.81	0.79	0.89
Medium-Level Learners	0.76	0.74	0.86
Low-Level Learners	0.72	0.69	0.83

## Data Availability

The data presented in this study are available upon request from the corresponding author.
